# Structure Based Discovery of Small Molecules to Regulate the Activity of Human Insulin Degrading Enzyme

**DOI:** 10.1371/journal.pone.0031787

**Published:** 2012-02-15

**Authors:** Bilal Çakir, Onur Dağliyan, Ezgi Dağyildiz, İbrahim Bariş, Ibrahim Halil Kavakli, Seda Kizilel, Metin Türkay

**Affiliations:** 1 Department of Chemical and Biological Engineering, Koç University, Sariyer, Istanbul, Turkey; 2 Department of Molecular Biology and Genetics, Koç University, Sariyer, Istanbul, Turkey; 3 Department of Industrial Engineering, Koç University, Sariyer, Istanbul, Turkey; University of South Florida College of Medicine, United States of America

## Abstract

**Background:**

Insulin-degrading enzyme (IDE) is an allosteric Zn^+2^ metalloprotease involved in the degradation of many peptides including amyloid-β, and insulin that play key roles in Alzheimer's disease (AD) and type 2 diabetes mellitus (T2DM), respectively. Therefore, the use of therapeutic agents that regulate the activity of IDE would be a viable approach towards generating pharmaceutical treatments for these diseases. Crystal structure of IDE revealed that N-terminal has an exosite which is ∼30 Å away from the catalytic region and serves as a regulation site by orientation of the substrates of IDE to the catalytic site. It is possible to find small molecules that bind to the exosite of IDE and enhance its proteolytic activity towards different substrates.

**Methodology/Principal Findings:**

In this study, we applied structure based drug design method combined with experimental methods to discover four novel molecules that enhance the activity of human IDE. The novel compounds, designated as D3, D4, D6, and D10 enhanced IDE mediated proteolysis of substrate V, insulin and amyloid-β, while enhanced degradation profiles were obtained towards substrate V and insulin in the presence of D10 only.

**Conclusion/Significance:**

This paper describes the first examples of a computer-aided discovery of IDE regulators, showing that *in vitro* and *in vivo* activation of this important enzyme with small molecules is possible.

## Introduction

Insulin degrading enzyme (IDE), an 113 kDa cytosolic metallopeptidase, found in bacteria, fungi, plants, and animals, is involved in the clearance of a variety of peptides including insulin, amyloid-β, glucagon, amylin, β-endorphin, insulin-like growth factor II (IGF-2), atrial natriuretic peptide (ANP), transforming growth factor α (TGF-α) [1], [2], [3]. IDE consists of two 56 kDa catalytic N- and C-terminal domains having four structurally homologous αβ roll domains [4]. These two N- and C-terminal domains, connected by a loop of 28 amino acid residues, constitute a large catalytic chamber where peptides smaller than 70 residues can fit. IDE can be in an open or closed state based on substrate-bound and substrate-free crystal structures of IDE [5], [6]. In the open state, substrates can diffuse in and out of the chamber, while free and bound substrates are degraded in the closed state. A unique feature of substrate recognition of IDE is that N-terminal of the IDE substrates make contact with a highly conserved region, named exosite, located ∼30 Å away from the catalytic center prior to cleavage [5]. This site allows proper positioning of the C-terminal end of peptides, and these peptides are subsequently directed towards the catalytic site.

There are several proposed mechanisms for the regulation of IDE activity [7], [8], [9]. One other possibility towards regulation of IDE activity is by small molecules, and it is possible to find small molecules that bind IDE and regulate its activity for stabilizing conformations or disrupting hydrogen bonding between N- and C- terminal halves. The first known small molecules that bind and activate IDE are ATP and other nucleotide polyphosphates [10], [11]. ATP is reported as an inhibitor of IDE [11]; however, interestingly it is found that it activates the degradation of shorter substrates by70 fold, higher than the wild type protein alone [12], [13].

The regulation of IDE function with small molecule organic compounds is becoming a very attractive strategy for the treatment of AD and T2DM. Cabrol *et al.* introduced two novel compounds that stimulated the proteolysis of only short peptides of IDE synergistically with ATP using high-throughput screening [12]. To our knowledge, these two molecules are the only small molecules that enhance the catalytic activity of IDE reported in the literature. In another study, a set of IDE peptide-inhibitors were designed to regulate the catabolism and activity of insulin. These resulting compounds were about 106 times more potent than existing inhibitors, and crystallographic analysis revealed that inhibitors stabilized IDE's “closed,” inactive state [13]. In a recent study by Tang and colleagues, a short peptide substrate bradykinin was discovered to increase the activity of IDE with selective binding to the exosite [6]. However, this peptide was found to have low affinity and high *K_m_* due to its failure to bind to the exosite and catalytic site simultaneously. The discovery of bradykinin as a low affinity IDE activator provided the knowledge basis for the design of molecules that may modulate the function of IDE. Consistent with this finding, we hypothesized that similar to bradykinin, binding of small molecules to the IDE exosite could play a regulatory role in substrate binding and subsequent IDE proteolysis.

In this study, we utilized structure based virtual screening approach to identify novel compounds that bind IDE exosite to enhance degradation of specific substrates by IDE. We performed virtual screening of over one million small molecular weight compounds, using the docking software Autodock 3.0.5. We identified 10 hit compounds that have high binding energies with the exosite of IDE, and further tested the effect of these compounds with fluorogenic assay using substrate V, insulin and amyloid-β. Three out of these 10 compounds, D3, D4 and D6, have been found to enhance the activity of the IDE towards insulin, substrate V and amyloid-β degradation, respectively, while the fourth compound, D10, enhanced the proteolysis of both substrate V and insulin by IDE. In addition, we show that degradation of insulin by IDE can be enhanced in a cellular setting, increasing the proteolysis of internalized insulin. In order to demonstrate further that these compounds indeed bind to the exosite of IDE, we used site-directed mutagenesis to synthesize IDE with mutations at the exosite, and tested those mutants to see whether such mutants have altered the effectiveness of our compounds. Our experimental analysis demonstrated that mutations of IDE exosite residues Gln363 and His332 to alanine resulted in lower initial and maximum rate of proteolysis of insulin by IDE. The novel compounds proposed in this study could serve as initiators for lead optimization studies, which could ultimately lead towards the design of compounds for the treatment of hyperinsulinemia and Alzheimer's disease.

## Results

### Analysis of Molecular Dynamics Simulation

We applied an energy minimization and molecular dynamics simulation for the refinement of atom coordinates of IDE (pdb id: 2G47) as described previously [14] . This procedure is required to have a 3D structure similar to IDE at physiological conditions before docking calculations in order to calculate binding energies at realistic conditions. The simulation showed a slight increment after minimization up to approximately 0.4 ns, and then no significant deviations for the rest of the simulation were observed, which demonstrated the convergence of RMSD and the stability of the given structure within 1.2 ns time period. The final structure at the end of the MD simulation was employed for molecular docking calculations.

### Virtual screening and detailed docking

The database of over 1,000,000 commercially available compounds was used for virtual screening as described previously [14] and the experimentally determined inhibition constant (IC_50_) values were shown to support computational predictions about the activity of the compounds found [15], [16]. The study of Malito et al. confirmed that binding of a short peptide substrate, bradykinin, to the exosite region of IDE enhanced the degradation of short peptide substrate [6]. Therefore, the exosite region was selected as the target region for computer-aided drug design. The free binding and docking energies of the selected candidates were in the range of −9 to 15 kcal/mol. The top-ranking compounds from the virtual screening were chosen for further detailed docking simulations.

Following the virtual screening, the selected compounds were subjected to detailed -docking calculations at the exosite with increased grid resolution (from 0.375 Å to 0.180 Å). The compounds with minimum binding energies were selected and then analyzed according to important characteristics such as docking positions, hydrogen bonding interactions with the exosite residues (336–342 and 359–363) and close proximity, van der Waals interactions, exposed surface area, and pocket occupancy. Consideration of Lipinski rules [17], and visual analysis of compounds in terms of compound conformations yielded a final number of 10 compounds. A list of the top 10 compounds with estimated binding and docking energies in both virtual screening and detailed docking as well as their interactions with the residues at the exosite are listed in Table 1 (see Figure S8 for molecular structure of the compounds).

**Table 1:** Binding free and docking energy scores of virtual screening and detailed docking for the potential hit compounds.

ID	Bind. Free Energy (kcal/mol)	Docking Energy (kcal/mol)	Interacting residues
**D1**	−11.01	−12.33	His^332^, His^336^, Gly^339^, Gly^361^, Asn^418^, Tyr^609^
**D2**	−11.76	−14.06	Gly^331^,His^332^, His^336^, Gly^339^, Gly^361^, Gln^363^, Tyr^609^
**D3**	−11.13	−13.11	His^336^, Gly^361^, Glu^453^, Tyr^609^
**D4**	−11.16	−13.15	His^335^, Gly^339^, Gly^361^, Gln^363^, Tyr^609^
**D5**	−12.99	−14.89	His^332^, His^335^, His^336^, Gly^361^
**D6**	−11.77	−14,23	His^332^, Gly^361^, Gln^363^
**D7**	−12.06	−15.14	Gly^331^, His^332^, His^335^, His^336^, Gly^339^, Gly^361^ Gln^363^
**D8**	−11.57	−13,95	His^332^, Gly^339^, Gln^363^, Tyr^609^
**D9**	−12.18	−15.26	Asn^329^,Gly^331^, His^332^, His^336^, Gly^361^, Gln^363^, Asn^418^
**D10**	−11.48	−11.11	His^332^, Gln^363^

### Computational analysis of the novel compounds

**Figure 1:**
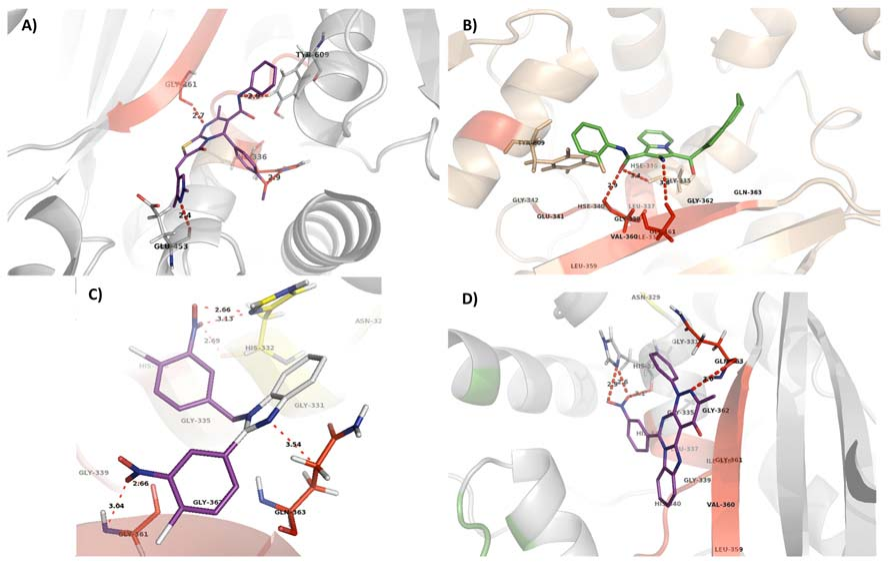
The interactions between compound D3 and the exosite of IDE. The compound is shown as purple sticks, amino acid residue belonging to the IDE exosite are shown as red sticks (Gly361) (A). The interactions between compound D4 and the exosite of IDE. The compound is shown as green sticks, amino acid residues belonging to the IDE exosite are shown as red sticks (Gly339, Val360, Gly361) (B). The interactions between compound D6 and the exosite of IDE. The compound is shown as purple sticks; amino acid residues belonging to the IDE exosite are shown as red sticks (Gly363, His332, Gly361) (C).The interactions between compound D10 and the exosite of IDE. The compound is shown as purple sticks; amino acid residues belonging to the IDE exosite are shown as red sticks (Gly363, His 332) (D).

The structures and conformation of compounds D3, D4, D6 and D10 at the IDE exosite, and their interactions with IDE residues and distances between interacting atoms are shown in Figure 1. The threshold value for hydrogen bonding interaction is considered as 3.5 Å; that is hydrogen bonding interaction is established if a distance between a hydrogen donor atom and a hydrogen acceptor atom is smaller than this threshold value as well as the angle between these atoms. As shown in Figure 1A, D3 interacts with the exosite residue His336 and Gly361 and also additional contacts were observed with Glu453 and Tyr609. D4 interacts with three exosite residues, Gly339, Gly361, Gln363 (Fig. 1B), and additional interactions were observed with His335 and Tyr609. As can be observed in Figure 1C, D6 creates interactions with Gly361, Gln363 at the exosite and additional interaction with His332 is also observed. Finally, D10 has interactions with Gln363 residue at the exosite and His332 as shown in Figure 1D.

It can be postulated that, binding of bradykinin at the exosite stimulates the switch from close to open state; however, the stability and duration of open state can be increased using more efficient compounds. The conformational change for IDE is required to enhance the catalytic activity due to its complex allosteric nature. It can be speculated that our lead compounds can enter into a partially opened chamber due to their small sizes; consequently interacting with exosite residues with similar mechanism as bradykinin. This may result in a conformational change of IDE by reducing its catalytic chamber size, and this shift may yield the open state which will subsequently allow substrates to interact with the catalytic site [6].

### Effect of novel compounds on the enzymatic activity of IDE

The 10 compounds given in Table 1 were subjected to further characterization and their effect was measured on the proteolytic activity of IDE using different substrates. Initially, activity assays were carried out at fixed 20 µM concentrations for all molecules to identify the effect of those compounds on IDE activity. IDE activity was quantified by monitoring the change in fluorescence for the proteolysis of fluorogenic-substrate V, FITC- insulin-bound antibody, and FAβB by recombinant human IDE purified from *E.coli* BL21 [6], [18], [19]. Analysis for the effect of compounds on IDE activity showed that, D3 increased the IDE-mediated insulin degradation by 72% (Fig. S1B), while D4 enhanced IDE-mediated substrate V and insulin degradation by 36% and 60%, respectively (Fig. S1A, B). Also, D6 increased the amyloid-β degradation by 20% (Fig. S1C). Further, D10 enhanced IDE-mediated FITC-insulin-mAb and substrate V degradation by 290% and 65%, respectively (Fig. S1A, B). Next, an EC_50_ value of each molecule that enhanced IDE activity was determined with a dose response assay, where specific activity of IDE was measured with different concentrations of the lead compounds and then the plot was fit to Sigmoidal Hill-4 parameter equation. Calculated EC_50_ values for D4 were found as 7 µM and 13 µM for substrate V, and insulin degradation, respectively (Fig. S2A, B). EC_50_ value for D3 on insulin degradation was approximately measured as 20 µM (Fig. S2C), whereas EC_50_ value for D6 on amyloid-β degradation was 2 µM (Fig. S2D). Furthermore, EC_50_ values for D10 were found as 13 µM and 12 µM for substrate V, and insulin degradation, respectively (Fig. S2E, F). Among all these four compounds, D10 seems to be the potent activator of IDE, when proteolysis of insulin is considered.

The effect of ATP on insulin degradation was further tested with compounds D3, D4 and D10, and we observed that IDE activity did not change significantly when proteolysis was carried out in the presence of 0.1 mM ATP (Fig. S3). Cabrol *et al.* found a synergetic effect of ATP with potential compounds; thereby in order to test the effect of ATP on IDE activity in the presence of activators, we, performed a fluorogenic assay with different concentrations of the novel compounds [12]. The novel compound, D10 enhanced the substrate V degradation by 60%, however this compound is more potent on insulin degradation with an increase in the IDE activity of 290% (Fig. S3C). Finally, ATP had no effect on amyloid-β degradation in presence of D6 (data not shown).

### Effect of Compounds on Initial Rate of Proteolysis by IDE

**Figure 2:**
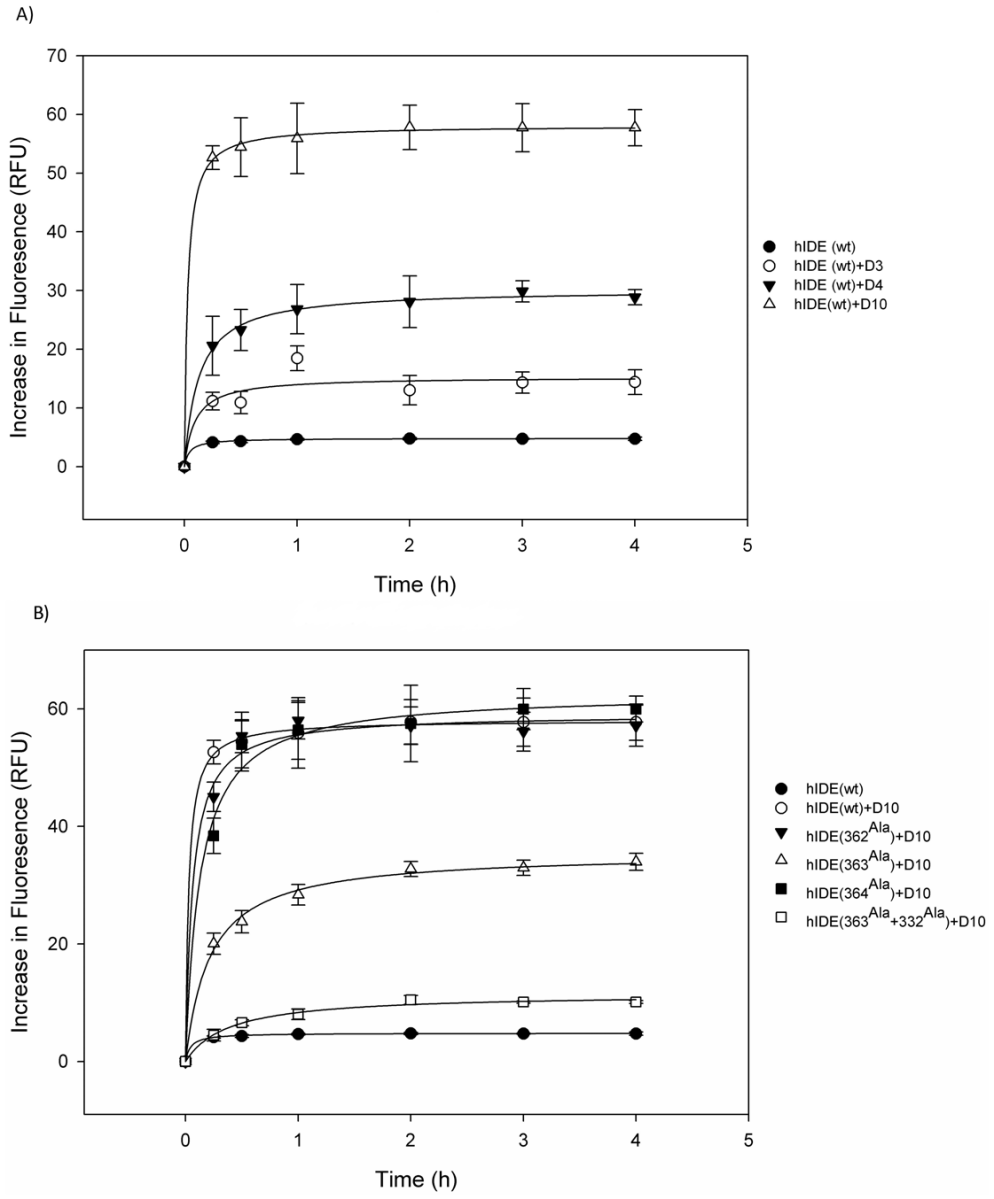
Measurement of initial insulin binding rates of IDE. Effects of different drugs (D3, D4, and D10) on initial binding rate of insulin (A). Initial insulin binding rates of mutant IDE in the presence of 20 µM D10 (B). Data are mean ±SEM for 3 independent experiments (p<0.0001).

In order to explore the effect of lead compounds on the initial activity of the IDE, we carried out time series experiments both in the presence and absence of the lead compounds. Among the lead compounds, only D10 increased the initial rate of substrate V degradation by about 2 fold (Fig. S4). On the other hand, D3, D4, and D10 significantly increased the initial rate of proteolysis activity of IDE on FITC-insulin bound with antibody by about, 7.5-, 12-, and 49-fold, respectively (Fig. 2A). D3, D4 and D10 did not have any effect on the proteolysis activity of IDE for amyloid-β (Fig. S1C). Furthermore, our results indicated that D6 accelerated the initial rate of amyloid-β proteolysis by IDE by a factor of 1.3 (Fig. S4B). It is observed that initial activation of enzyme increased even at very low concentrations of lead compounds (Fig. 2A). Interaction of these compounds with exosite residues similar to bradykinin might be stabilizing the enzyme conformation in its active state. As a result, interaction of the lead compounds with IDE may change the conformation of IDE by reducing the size of the catalytic chamber, and this shift may promote the open state of the enzyme. This mechanism was observed for bradykinin in Malito *et al.* [6] Structurally stabilized IDE in open state may subsequently allow substrates to interact with the catalytic site in an efficient manner and result in sharp increases in initial rates (V_o_) even in the presences of low concentration of the compound.

### Verification of exosite as the binding site by lead compounds via directed mutagenenesis

Since D10 has been found as the strongest activator of IDE towards the proteolysis of insulin, we analyzed the binding site of the compound D10 for the further analysis. According to the computational results, D10 binds to Gln363 at the IDE exosite and His332 (Fig. 1D). We carried out site-directed mutagenesis by replacing these amino acids into Ala in order to verify that D10 indeed binds to this region via these amino acids. We first mutated Gln363 to Ala and then expressed and purified mutant protein through nickel resin. The effect of D10 on the proteolytic activity of mutant IDE (Gln363Ala) was measured towards FITC-insulin-mAb degradation. Enzymatic *in vitro* assay showed that enhanced activation in the presence of D10 was reduced by 10-fold with mutant IDE (Gln363Ala). The activation was about 49-fold in the presence of D10 with wild type IDE (Fig. 2B). Although mutant IDE activity was less responsive to D10 compound compared to wild type IDE, it appeared as if D10 were interacting with IDE through His322 and could enhance the activity of mutant IDE. Therefore, we generated a double mutant IDE (Gln363Ala, His332Ala) in order to eliminate the binding of D10 to the exosite region completely. Analysis of double mutant demonstrated that D10 could not bind to IDE exosite (Fig. 2B). This was verified by enzymatic assay which resulted in similar initial rate of proteolysis for both double mutant and wild type IDE (Fig. 2B). If Gly363 were in fact one of the amino acids that interacts with D10, one would expect to see amino acids (Gly 362 and Lys 364) that are adjacent to the Gly363 would not contribute to the binding of the D10. Therefore we mutated adjacent amino acids (Gly 362 and Lys 364) to Ala and showed that D10 compound could in fact interact with Gly363. The initial rate of proteolytic activity of double mutant IDE (Gln363Ala, His332Ala) is comparable to wild type IDE, where D10 increased the activity of IDE over 49-fold as can be observed from Figure 2B. These results suggest that D10 binds to IDE through Gly363 at the exosite, and His 332, and in turn can increase the catalytic activity in a specific manner. Furthermore, we have carried out competition assay with bradykinin in the presence of D10 in order to demonstrate further that D10 binds to the exosite region of IDE. Assay is performed with three different concentrations of bradykinin (2.1, 4.2 and 8.4 mM) and with varying concentrations of D10. We chose 2.1 mM and 8.4 mM concentrations as minimum and maximum amounts of bradykinin in this experiment, as *K_m_* value for bradykinin was measured as 4.2 mM in a previous study (Fig. S6) [6]. This experiment demonstrated that degradation of insulin in the presence of D10 was decreased by about 50% when there was bradykinin in the proteolytic medium, even at high concentrations of bradykinin (8.4 mM) (Fig. S6). This could be attributed to the competition of D10 with bradykinin to access to the exosite residues of His332 and Gln363 (Table 1).

### Cell Viability Test

We characterized the activity of novel compounds in a cellular assay to establish whether there were any cytotoxic effects. We used an established MTT (3-(4,5-dimethylthiazolyl-2)-2, 5-diphenyltetrazolium bromide or thiazolyl blue) method using HELA cells to determine whether the small molecule affects cell viability [20]. In this method, a purple formazan dye forms as a result of the cleavage of the yellow tetrazolium MTT within metabolically active cells. The resulting precipitate of the intracellular formazan can be dissolved in a detergent solution and quantified spectrophotometrically at 595 nm. We measured the cytotoxicity by incubating HELA cells in the presence of novel compounds at concentrations ranging from 1 to 100 µM for 24 h. Cell viability was measured using the MTT method after 12 h culture. The extent of cell death was expressed relative to a control containing DMSO. We found that D4 did not affect viability significantly from a concentration of 2 µM to a concentration as high as 100 µM at which cells remained 60% viable (Fig. S5B). We also observed that approximately 85% of cells remained viable at a concentration of 7.5 µM, which is close to the EC_50_ value of D4. The other novel compounds, D3 and D10 did not demonstrate any toxicity at high concentrations (Fig. S5A, D). We observed that D6 showed toxicity at higher concentrations (Fig. S5C), while D6 is not toxic at its EC_50_ value of 2 µM. These results indicate that lead compounds are not toxic to mammalian cells. Therefore, as a next step we tested the effects of lead compounds on insulin catabolism using HeLa cells.

### Cellular-based degradation of insulin

**Figure 3:**
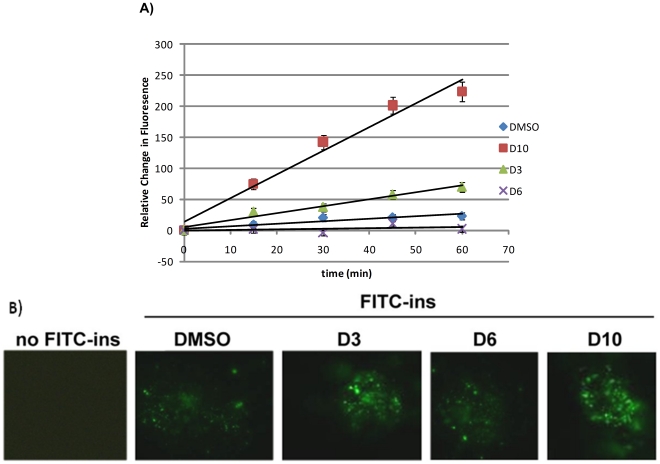
Effects of IDE activators on insulin catabolism in Hela cells. Compound D10 and D3 show ∼%240 and %65 increases respectively in the magnitude of IDE activity for insulin degradation in HeLa cells (A). D6 does not change insulin degradation as expected (A). Representative images of live HeLa cells pre-loaded with FITC-insulin and imaged at various drugs (20 µM) and absence of drugs (B). Data are mean ±SEM for 3 independent experiments (p<0.0002).

Biochemical analysis indicated that lead compounds increased the activity of IDE enzyme towards different substrates, predominantly towards insulin. In order to demonstrate further that these molecules can display activity *in vivo* conditions, we examined the effects of lead compounds on insulin catabolism in cultured cells. It was shown that HeLa cells express insulin receptor, and thus take FITC-labeled insulin inside and degrade it intracellularly [13], [21], [22]. First, FITC-insulin was added to the culture that contains HeLa cells and incubated for 2 hours in order to allow for sufficient uptake of FITC-insulin by HeLa cells. In order to ensure that FITC-labeled insulin is translocating into the cells through insulin uptake receptor, and that cytoplasmic IDE can degrade FITC- insulin, we conducted live cell imaging of HeLa cells, and recorded fluorescence. At various time points compounds, D3, D6, and D10, were added; and cells were washed with Hank's balanced salt solution (HBSS), the these cells were collected and lysed with appropriate buffer. After centrifugation, cell free extracts were used to measure the change in fluorescence by luminometer. The cells that were treated with D10 and D3 displayed increases in the intensity of fluorescence. Consistently, we observed higher fluorescence signal from D10 and D3 treated cells suggesting higher FITC-insulin degradation by cytosolic IDE (Fig. 3A). Compound D10 increased *in vivo* proteolysis of FITC- insulin by about 32 fold with HeLa cells compared to control group (Fig. 3A). However, for the case with compound D6, which was used as a control, fluorescence intensity did not change significantly compared to the samples treated with D10 and D3. These findings are consistent with the data obtained by *in vitro* assay, showing enhanced degradation of insulin by D10 and D3 (Fig. 3A). Notably, D6 had no effect on insulin catabolism as expected (Fig. 3A). Figure 3B shows fluorescence microscope images of HeLa cells ten minutes after the addition of compounds D3, D6, D10, as well as images of HeLa cells treated with only DMSO and no-FITC insulin as controls. This figure further verifies the significant increase in fluorescence observed in the presence of compound D10, which is consistent with our *in vitro* assay (Fig. 3B).

## Discussion

Virtual screening has become one of the major components in the drug discovery approach within the last few years [23]. When 3D structure of the protein is known, structure based screening is preferred, whereas ligand based screening is utilized when experimental inhibition/activation data is available in the literature [24]. The presence of the crystal structure of IDE makes structure based drug design a viable approach to find novel molecules that could regulate overall activity of the IDE. Here, we employed structure based virtual screening, enzymologic characterization, cellular based assay to discover potent compounds that regulate IDE activity. The hit rate in experimental high-throughput screening (HTS) efforts of drug-like compounds varies from 0.01 to 1% [25]. Cerchietti *et al.* achieved a 10% hit rate using computer-aided drug design to identify BCL6 inhibitors [26]. In another study, novel inhibitors of protein tyrosine phosphatase-1B were identified with a hit rate of 34.8% (127 potent compounds out of 365 selected molecules) [27]. Here, we achieved 40% (4 out of 10 tested) of hit rate using structure based drug discovery approach, which is a faster, more economic and efficient strategy compared to other HTS or experimental screening techniques. We utilized structure based virtual screening approach to identify four compounds that bind to IDE exosite, and have potential to enhance cleavage of specific substrates by IDE. Three compounds, D3, D4 and D6, have been found to enhance the activity of the IDE towards insulin, substrate V and amyloid-β degradation, respectively, while compound D10, enhanced the proteolysis of both substrate V and insulin by IDE. A recent study by Cabrol et al. showed enhanced degradation of amyloid-β by two compounds, Ia1 and Ia2, in the presence of short peptide substrates and ATP, while in other study by Leissring et al. [12] a rational design approach based on analysis of combinatorial peptide mixtures was used to develop IDE inhibitors [12], [13]. To our knowledge, the compounds discovered in this study are the first potential lead compounds that enhance IDE activity independently of ATP or small IDE substrates. The optimization of the structure of small molecular weight organic compounds identified in this paper and further *in vivo* characterization may make it possible to design molecules with therapeutic utility for hyperinsulinemia, type 2 diabetes and Alzheimer's disease.

The most significant novelty that these compounds have is that they are the only known selective and independent activators of IDE. Specifically, to our knowledge compound D10 is the only potent small molecule discovered to increase proteolysis of insulin by IDE. The distinctive IDE structure made it difficult to discover compounds with therapeutic value, as IDE has two distinct domains with C and N terminal halves, and can only degrade its substrates when the protease is in closed configuration. Previous study by Malito *et al.* highlighted the complexity involved in the substrate binding and recognition mechanism of IDE, and demonstrated that IDE uses its exosite to anchor the N-terminus of its substrates which are then cleaved through a stochastic process at the catalytic site [6]. Previous crystal structures of bradykinin with IDE also showed that bradykinin can only bind to the exosite but could not reach to the catalytic site. This observation also suggested that bradykinin could play a regulatory role to enhance substrate binding and degradation by reducing the entropy of short peptides in the chamber. The lead compounds that are presented in this paper may enhance binding of different substrates to the exosite and then direct substrates to the catalytic site for further proteolysis. Our site directed mutagenesis and competition assay at the IDE exosite results with these novel compounds also suggest that binding of compounds to the exosite indeed occurs, and that binding to the exosite is critical for enhancing substrate specific degradation by IDE. It is established that catalytic rate of IDE is affected by flexibility of substrates as well as ability of substrates to gain access to the catalytic chamber. Furthermore, we observed that bradykinin competed with compound D10 for the binding to the IDE exosite. These results suggest that the compounds enter into the partially opened catalytic chamber during IDE conformational switch, and to direct substrates towards the catalytic site resulting in enhanced catalysis for specific substrates.

Various studies have shown that ATP enhances the IDE activity of some substrates and it sometimes plays a synergistic role with the drug-like compounds [12], [13]. We observed that the activity of IDE for insulin degradation did not change in the presence of ATP. The concentration dependent D3, D4 and D10 experiments were repeated in the absence and presence of ATP, and the activity of IDE showed the same trends in these conditions. Song *et al.* showed that nucleotides have no effects on the hydrolysis of the physiological substrates insulin and amyloid-β peptide [10]. We also observed that ATP has no effect on the insulin catabolism in the presence of D3, D4, and D10 in our experiments (see Fig. S3).

The most significant impact of the compounds reported in this paper from a biomedical point of view is that these compounds have the potential to have medicinal value on insulin signaling. IDE activators with desirable pharmacokinetic properties may be significant for diabetes, as it is questionable to combat with diabetes or hyperinsulinemia through genetic mutations of IDE gene [28]. For example, IDE knockout mice have been shown to develop diabetic phenotype, whereas overexpression of IDE in Alzheimer's disease mouse models have been shown to completely eliminate amyloid plaque formation and downstream cytopathology [29], [30]. In contrast to the permanent lifelong activation of IDE, drugs that only transiently enhance IDE activity for specific substrates may be expected to improve glycemic control. As a result, the goal of enhancing amyloid -β or insulin degradation by pharmacological compounds has been regarded as best, but a challenging objective due to the unusual properties of this enzyme.

Finally, the activators that are introduced in this study may function as important tools to manipulate IDE experimentally. The use of activators might also be important for experimental and clinical settings. Further, these compounds would be useful to address various questions related to the chemical biology of IDE. Our cellular based assay demonstrated that compounds that enhance degradation of insulin, D3, D6, and D10, not only enhance degradation of insulin *in vitro* and *in vivo* but they are also permeable through cell membrane which may allow for evaluation of their roles in animal models for diseases such as hyperinsulinemia, diabetes, and Alzheimer's.

## Materials and Methods

### Materials

cDNA of human insulin degrading enzyme (IDE) was obtained from Prof. Richard A. Roth from Stanford University. Substrate V was purchased from R&D systems, Inc. (Minneapolis, MN), FITC and 1, 10- phenanthroline from Sigma, fluorescein-Aβ-(1–40)-Lys-biotin (FAβB) and bradykinin from Anaspec Corp (San Jose, CA), Neutravidin- coated agarose beads from Thermo Scientific and mouse anti human insulin from AbD Serotec.

### Molecular dynamics simulation

The coordinate of the initial structure was obtained from the Protein Data Bank (PDB id: 2G47) [4]. The reported structure was a catalytically inactive IDE mutant IDE-E111Q in complex with amyloid-β peptide (Aβ 1–40) at 2.1 Å . MD simulations were carried out by using the NAMD software, version 2.6 with the PARAM22 version of the CHARMM force field [31], [32]. The protein was solvated in a rectangular box including TIP3P water molecules and counter ions. First, the system was minimized through 10000 steps by keeping the backbone fixed, and then backbone atoms were relaxed through 10000 steps. Next, the system was heated to 310 K with 10 K increments (10 ps simulation at each increment).

After the equilibration of the system, a molecular dynamics simulation was carried out with constant temperature (310 K) and pressure control using the Langevin piston method. The time-step of the simulation was set to be 2 fs and, the bonded interactions, the van der Waals interactions (12 A cut-off), and the long-range electrostatic interactions with particle-mesh Ewald (PME) were included in the calculations to define the forces acting on the system. The damping coefficient was set to be 5 ps-1 using Langevin dynamics to handle pressure control. The simulation was carried out for 1.2 ns, and the final stable structure was used later in the docking calculations.

### Molecular Docking

For small docking calculations docking software AutoDock 3.05 which was available for public access was employed [33]. This version of Autodock predicts the optimal conformations of the receptor-ligand complex and report binding affinity scores by assuming a structure model with a rigid receptor (protein) and a flexible ligand. The scoring function of Autodock consists of electrostatic, Lennard-Jones, hydrogen bond, solvation, and torsional entropy terms. Lamarckian genetic algorithm (LGA) was employed for the ligand conformational search. The residues 336–342 and 359–363 of IDE were taken as the targets for docking, since this region was defined to be located in the exosite of IDE [6].

### Construction of expression plasmid for IDE protein

cDNA of hIDE was PCR amplified with primer sets that contains unique restriction enzyme sites (XhoI and NotI) and hexa-histidine tag (6×His). The primers that have been used for amplification of hIDE cDNA are forward primer: 5′-cttgcggccgcaatgcggtaccggtaccggctagcgtg-′3 and reverse primer: 5′- gtgctcgaggagttttgcagccatgaa-′3. PCR reaction is performed in total volume of 50 µl containing 100 ng of plasmid, 40 pmol of each primer, 0.2 mM dNTPs, and 2 unit of Taq DNA polymerase. 33 cycles of amplification reaction is performed with the conditions of 95°C for 30 s, 55°C for 30 s and 72°C for 3 min. The reaction mixture is kept at 95°C for 4 min before the first cycle, and after the 33 rd cycle additional extension period is applied at 72°C for 10 min. Then, PCR products were purified through the gel extraction method and digested with Xho I and Not I enzymes along with pET21-b bacterial expression vector. Then, cDNA of hIDE were subcloned into pET21-b according to the Maniatis et al. and named as pET21b-hIDE [34].

### Site Directed Mutagenesis

Site-directed mutations of the specified hot spot residues were introduced to hIDE by PCR as previously described using appropriate plasmid [35]. The presence of the specific mutations was verified by DNA sequencing at Burc Laboratory (Istanbul, Turkey).

### Expression and purification of IDE protein

pET21b-hIDE was transferred to *E.coli* BL21 cells for expression of recombinant hIDE. Once cells reached to OD A600 0.6–0.8, IPTG was added to a final concentration of 400 µM and hIDE was expressed at 37°C for 3 h. Next the cells were harvested and sonicated in a lysis buffer which was composed of 50 mM NaH_2_PO4, 300 mM NaCl, 10 mM imidazole, 100 mM PMSF, 50 mM protease inhibitor cocktail and 0.1 mg/ml lysozyme at a pH of 8.0. The soluble protein fraction was separated by centrifugation at 9000× g for 30 min, and filtered through a Ni-NTA column. After rinsing with 20 mM imidazole, the histidine-tagged enzyme was eluted by NaCl and 250 mM imidazole buffer. The purified IDE was concentrated using a dialysis tubing cellulose membrane (MWCO 12000 Da) and stored at −80°C in a storage buffered as mentioned previously [36]. The concentration of the IDE protein was measured as 0.02 mg/ml. The integrity of hIDE was characterized by electrophoretic separation and followed by Western-Blot. Proteins using anti-His IgG and membrane were visualized by the ECL plus system.

### Degradation of Substrate V by IDE

To assess the utility of these novel compounds as chemical regulators for IDE activity, we used fluorescence assay with fluorogenic substrate V (7-methoxycoumarin-4-yl-acetyl-NPPGFSAFK-2, 4-dinitrophenyl). Reaction was carried out in the presence of 105 µl of 10 µl of 40 µM substrate V; 10 µl of 0.02 mg/ml of IDE in 83 µl of 30 mM KH_2_PO_4_ at pH of 7.3, and various concentrations (0–35 µM) compounds and also with various time intervals (0–4 hours). The reaction mixture was incubated at 37°C for 2.5 h. The hydrolysis of substrate V was monitored using a Tecan Safire2 microplate reader with excitation and emission wavelengths set at 300 and 395 nm, respectively.

### Binding of monoclonal antibody (mAb) to FITC-insulin

In order to measure degradation of insulin by IDE, FITC-insulin is bound to the monoclonal mouse anti-human-insulin, so that the rotation of free FITC and possible changes in fluorescence polarization could be prevented. We performed enzymatic assays, using only FITC-insulin bound antibody as a background fluorescence and checked the effects of compounds on the cleavage of FITC-insulin-mAb. FITC-insulin is bound to mouse anti human insulin antibody in PBS at 37°C for 1 hour, which is higher than the optimum time required to obtain maximum binding of insulin-FITC conjugate and mAb [37]. It is observed that 400 nM mAb completely binds to 50 nM FITC-insulin (Fig. S7). Since molecular weights of FITC-insulin and mAb are about 5.8 kDa and 60 kDa respectively, we observed very slight shift in the gel as expected. In figure S7, it is observed in lane 4 that 50 nM (100 ng) insulin is completely bound to 400 nM mAb in lane 4 (Fig. S7). Lane 1 represents 400 nM antibody, lane 2 shows 100 nM insulin, and lane 3 expresses the binding between 400 nM antibody and 100 nM insulin. The binding between insulin and mAb is shown by performing (%5 and %15) native polyacrylamide gel electrophoresis.

### Degradation of FITC-insulin-mAb by IDE

The utility of these novel compounds were tested on degradation of insulin by IDE. First insulin was labeled with FITC as described previously [37]. Briefly, 500 mg insulin dissolved in 1.5 ml of 0.1 M Na-carbonate, at pH 7, and 0.2 mM EDTA. Then, 4.5 mg FITC was dissolved in 200 µl acetone and was added dropwise to previous solution at room temperature for 20 h ([FITC]∶[insulin], 3∶1). Finally, unreacted FITC was removed by dialysis and the FITC-insulin was stored at −20°C. The proteolysis of insulin by IDE was monitored by measuring the changes in fluorescence, where the reaction was carried out with 100 nM of FITC- insulin-mAb and IDE (80 ng) in 100 µl KH_2_PO_4_ buffer at a pH of 7.3. Reaction was carried out at 37°C for different time intervals (0–4 hours). The increase in fluorescence was monitored by using Fluoroskan Ascent FL microplate reader (Thermo).

### FAβB degradation assay

This assay was performed as follows: 10 µl of 50 nM of FAβB was dissolved in a buffer which consisted of 50 mM HEPES, 100 mM NaCl, % 0,05 (v/v) bovine serum albumin, at a pH of 7.4. The reaction was initiated in a total volume of 100 µl with 20 µM drugs by adding 20 µl of 0.02 mg/ml of IDE for 2.5 h at 37°C. The reaction was stopped by adding 2 µl of 100 mM of protease inhibitor (1, 10-phenanthroline). Uncleaved FAβB was precipitated with Neutravidin- coated agarose beads by gentle rocking for 30 min. and centrifugation for 10 min at 14000 g. The supernatant was transferred to the black 96 well plates and increase in fluorescence (488 excitation, 525 emission) was measured with a multi-label plate reader.

### Enzymatic competition assay

The effect of the bradykinin on the clearance of insulin was measured in the presence of compound D10. Competition reactions were carried out at 37°C by mixing 90 µl of 10 nM FITC insulin in 50 mM potassium phosphate buffer (pH 7.3) and different concentrations of bradykinin (2.1 mM, 4.2 mM and 8.4 mM). Various concentrations of D10 was added (0–50 µM) and reaction was initiated with the addition of 80 ng IDE. The fluorescence increase was monitored by using Fluoroskan Ascent FL microplate reader (Thermo).

### Kinetic analysis

The effect of the lead compounds on the initial rate of proteolysis of IDE was determined by measurement in fluorescence with time both in the presence and absence of the lead compounds. The kinetic data for IDE-mediated insulin, substrate V and amyloid-β degradation were plotted and fitted using SigmaPlot software (Sigmoidal, Hill, 4 parameter). For the initial rate calculations, kinetic data were fitted using SigmaPlot software (Hyperbola; Single Rectangular I, 3 Parameter).

### Cell -based degradation of insulin

Cells were cultured at 37°C under a humidified atmosphere of 5% CO_2_ in medium supplemented with 10% fetal bovine serum. Then, FITC-insulin was added to cell medium and incubated for 2 h at 37°C. Next, compounds were loaded at different time intervals separately. Cells were washed with Hank's balanced salt solution (HBSS), then disrupted with 1× passive lysis buffer. Fluorescence was measured with a luminescence multi-label plate leader, and changes in fluorescence was imaged by fluorescence microscope at Burc Laboratory (Istanbul, Turkey).

### MTT assay for evaluating cell viability

The cytotoxicity assay was performed using human cervical cancer HeLa cells. Cells were cultured at 37°C under a humidified atmosphere of 5% CO_2_ in medium supplemented with 10% fetal bovine serum and dispersed in replicate 96- well plates with 2.5×10^4^ cells/well. Compounds were then added. After 24 hours exposure to the chemical compounds, the cell viability was determined by 3-[4,5-dimethylthiazol-2-yl]-2,5-diphenpyltetrazolium bromide (MTT) assay [38]. The optical density (OD) of the wells was determined using ELISA plate reader at a test wavelength of 600 nm and a reference wavelength of 630 nm. Each measurement was carried out in triplicate.

## Supporting Information

Figure S1 The effect of selected compounds on the proteolytic activity of IDE. Compound D4 and D10 show ∼%36 and %60 increase respectively in the magnitude of IDE activity for substrate V degradation (*p<0.0006) (A). Compound D3 , D4 and D10 show ∼72%, 60% and %275 increase respectively in the magnitude of insulin binding (*p<0.02, **p<0.003, ***p<0.0003) (B). Compound D6 increases the IDE-mediated amyloid-β degradation ∼20% (*p<0.0002) (C). Data are mean ±SEM for 3 independent experiments.
(JPG)

Figure S2 Measurement of the increase of IDE activity for substrate V (A), and insulin (B) degradation in the presence of D4 at concentrations ranging from 0.02 to 40 µM. Increase in IDE activity for insulin degradation in the presence of D3 (C). Increase in IDE activity for amyloid-β degradation in the presence of D6 (D). Increase in IDE activity for substrate V (E), and insulin (F) degradation in the presence of D10.Data are mean ±SEM for 3 independent experiments (p<0.0001).
(JPG)

Figure S3 The effect of compound D3 (**A**), D4 (**B**), D10 (**C**) on IDE mediated hydrolysis of insulin when tested in the presence and absence of ATP (0.1 mM). Data are mean ±SEM for 3 independent experiments. Data are mean ±SEM for 3 independent experiments (p<0.0001).
(JPG)

Figure S4 Measurement of initial rates of IDE in the catabolism of substrate V (A) and Amyloid-β (B) in the presence of D4, D6, or D10.Data are mean ±SEM for 6 independent experiments (p<0.0001).
(JPG)

Figure S5 Measurement of the viability of HELA cells in the presence of ranging concentrations of D3 (**A**), D4 (**B**), D6 (**C**) and D10 (**D**)from 1 to 100 µM. Data are mean ±SEM for 6 independent experiments (p<0.00001).
(JPG)

Figure S6 Competition of bradykinin with compound D10. Degradation of insulin in the presence of D10 is decreased by about 50% when there was bradykinin in the proteolytic solution.
(JPG)

Figure S7 Binding of monoclonal antibody (mAb) to insulin. Lane 1: 400 nM mAb, lane 2: 400 ng insulin, lane 3: 400 nM mAb and 400 ng insulin, lane 4: 400 nM mAb and 200 ng insulin, lane 5: 1 mg/ml BSA.
(TIF)

Figure S8 Molecular structure of the compounds.
(PDF)
