# Supplementary figures and images for: Structure Based Discovery of Small Molecules to Regulate the Activity of Human Insulin Degrading Enzyme
Source: PLoS One. 2012 Feb 15;7(2):e31787. doi: 10.1371/journal.pone.0031787 (PMC3280214; doi:10.1371/journal.pone.0031787)

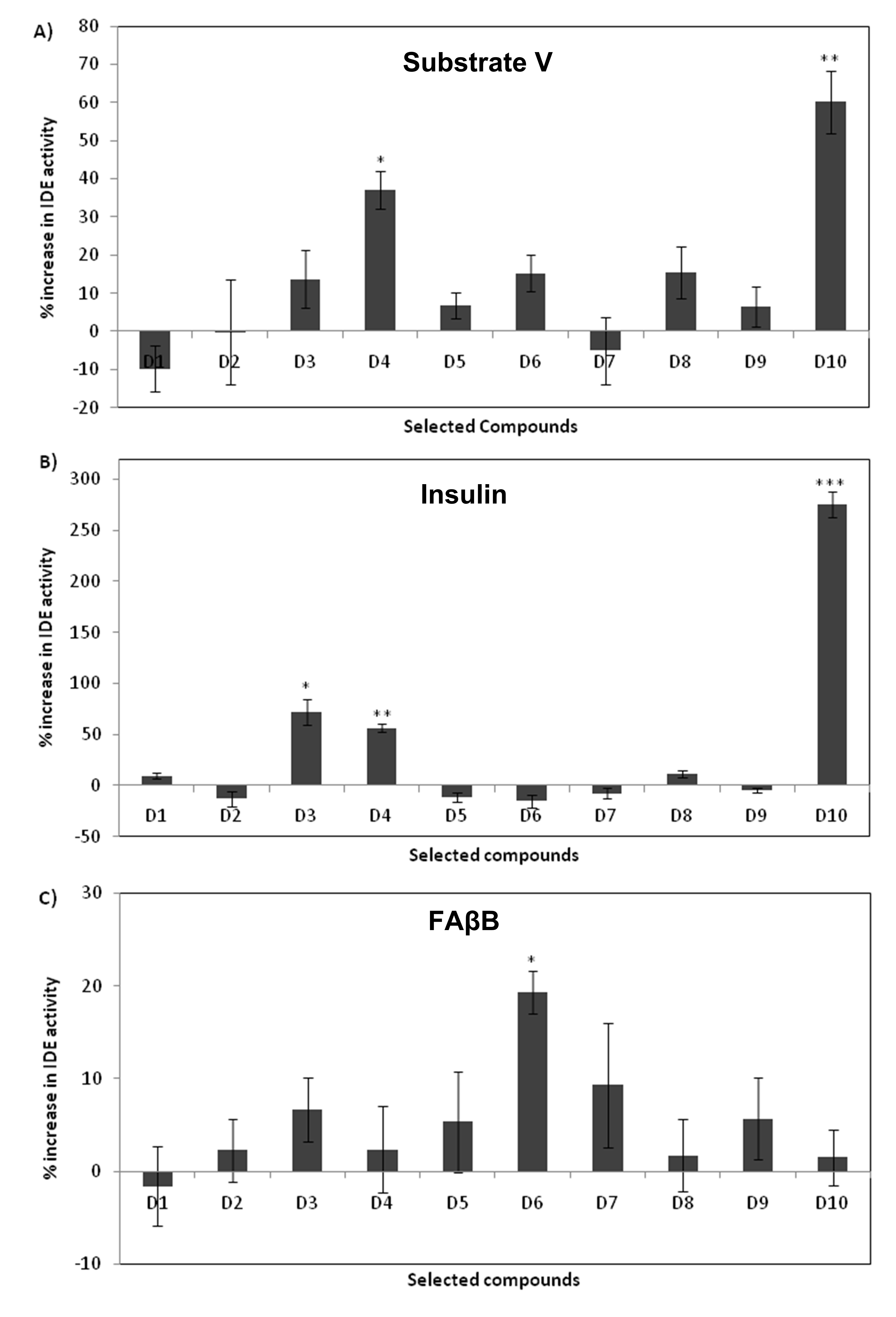

Supplement: Figure S1 — The effect of selected compounds on the proteolytic activity of IDE. Compound D4 and D10 show ∼%36 and %60 increase respectively in the magnitude of IDE activity for substrate V degradation (*p<0.0006) (A). Compound D3 , D4 and D10 show ∼72%, 60% and %275 increase respectively in the magnitude of insulin binding (*p<0.02, **p<0.003, ***p<0.0003) (B). Compound D6 increases the IDE-mediated amyloid-β degradation ∼20% (*p<0.0002) (C). Data are mean ±SEM for 3 independent experiments. (JPG) [file pone.0031787.s001.jpg]

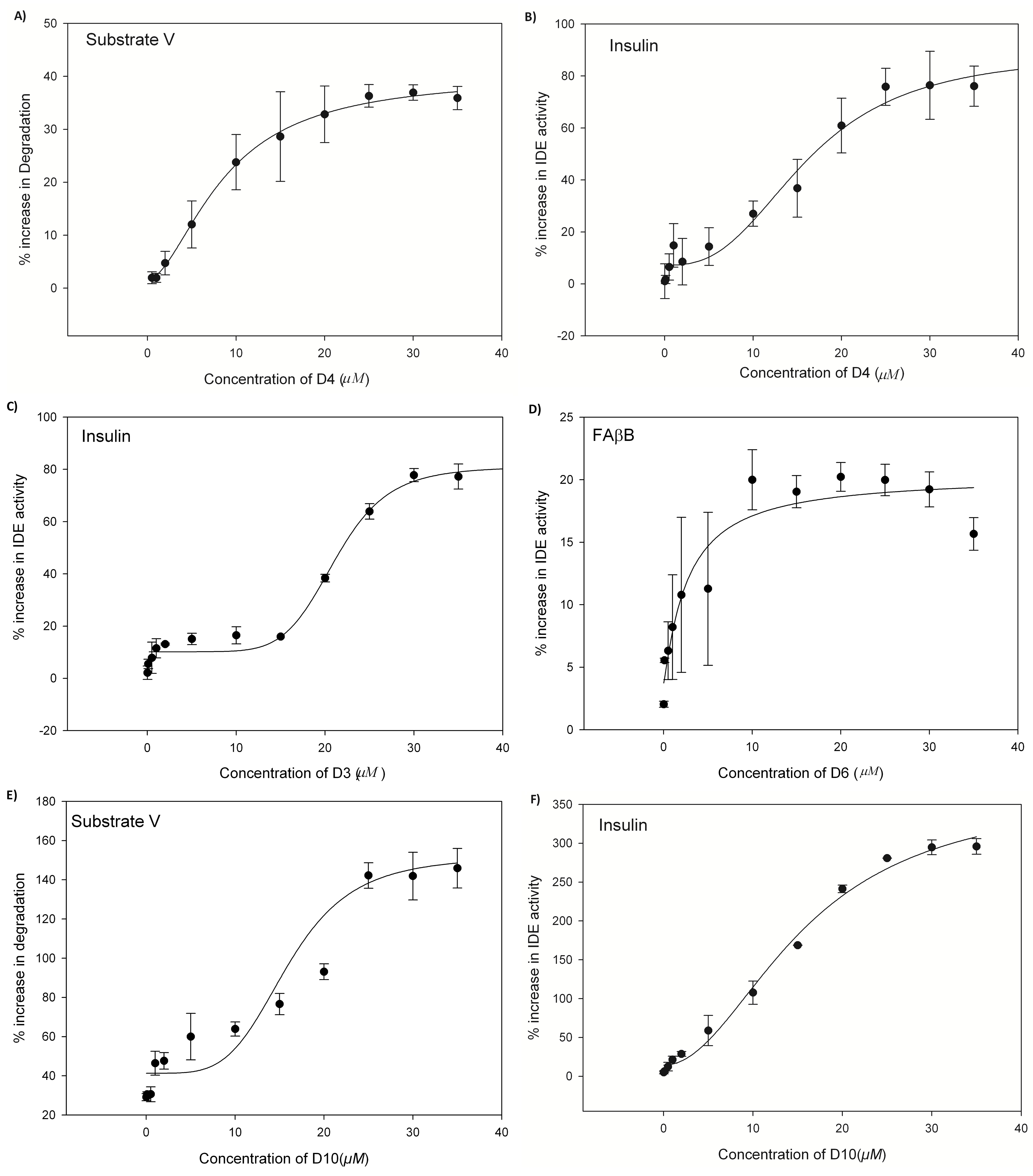

Supplement: Figure S2 — Measurement of the increase of IDE activity for substrate V (A), and insulin (B) degradation in the presence of D4 at concentrations ranging from 0.02 to 40 µM. Increase in IDE activity for insulin degradation in the presence of D3 (C). Increase in IDE activity for amyloid-β degradation in the presence of D6 (D). Increase in IDE activity for substrate V (E), and insulin (F) degradation in the presence of D10.Data are mean ±SEM for 3 independent experiments (p<0.0001). (JPG) [file pone.0031787.s002.jpg]

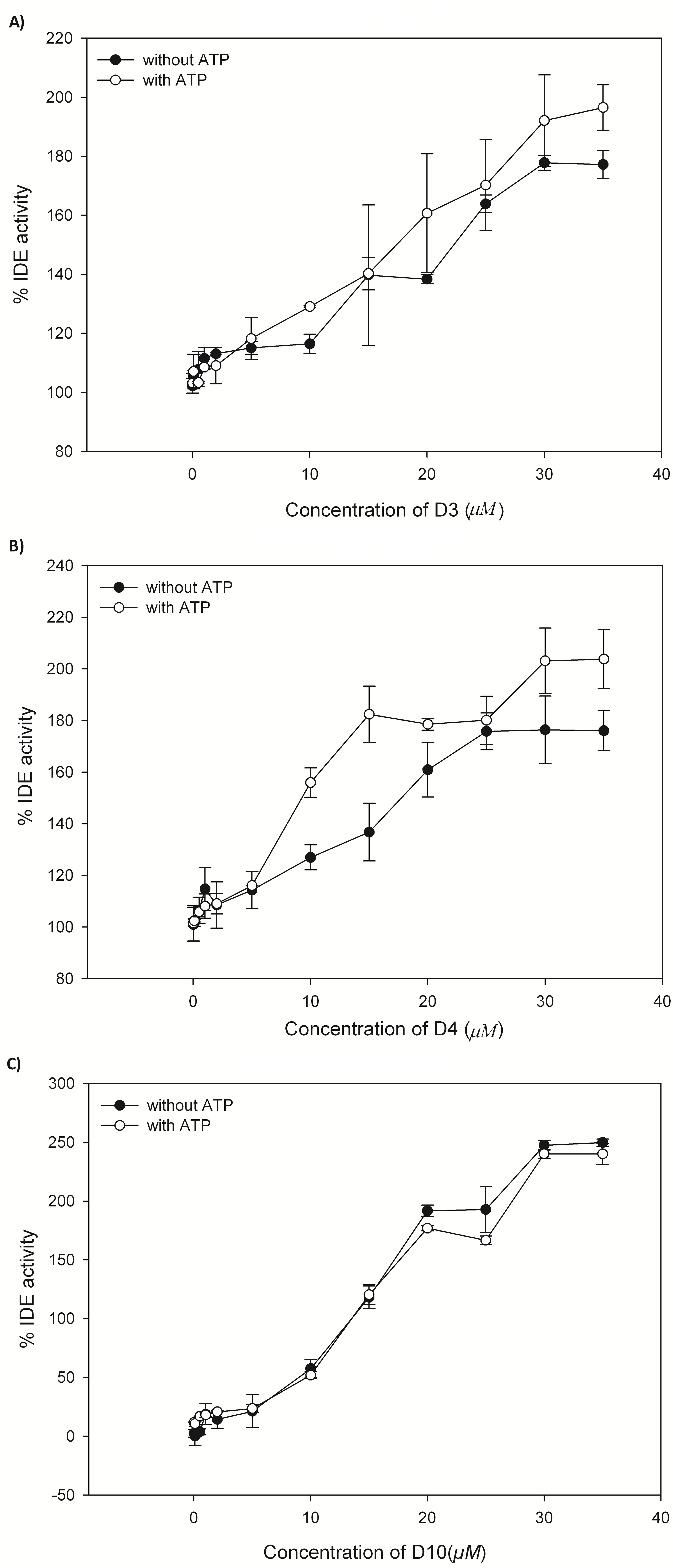

Supplement: Figure S3 — The effect of compound D3 (A), D4 (B), D10 (C) on IDE mediated hydrolysis of insulin when tested in the presence and absence of ATP (0.1 mM). Data are mean ±SEM for 3 independent experiments. Data are mean ±SEM for 3 independent experiments (p<0.0001). (JPG) [file pone.0031787.s003.jpg]

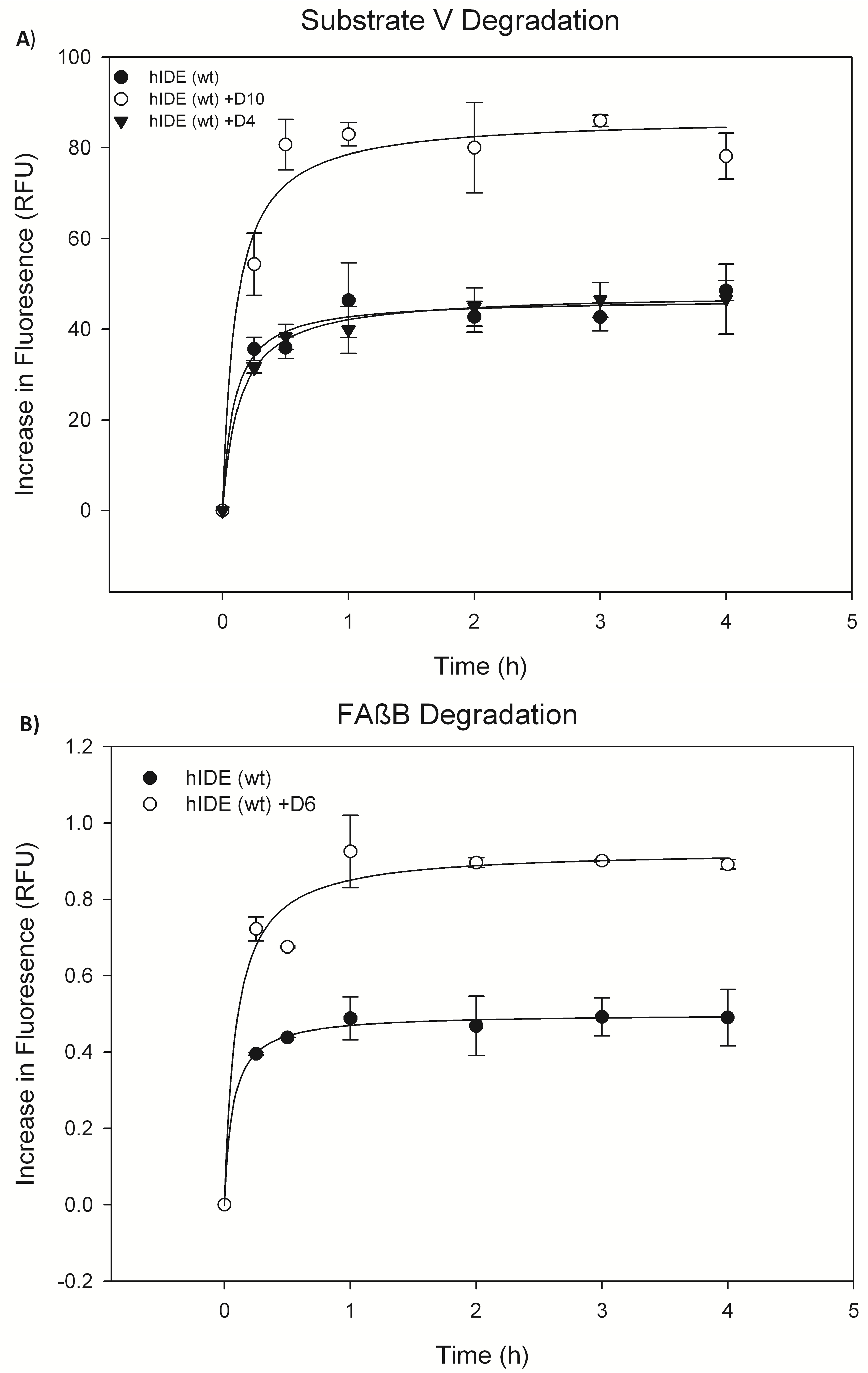

Supplement: Figure S4 — Measurement of initial rates of IDE in the catabolism of substrate V (A) and Amyloid-β (B) in the presence of D4, D6, or D10.Data are mean ±SEM for 6 independent experiments (p<0.0001). (JPG) [file pone.0031787.s004.jpg]

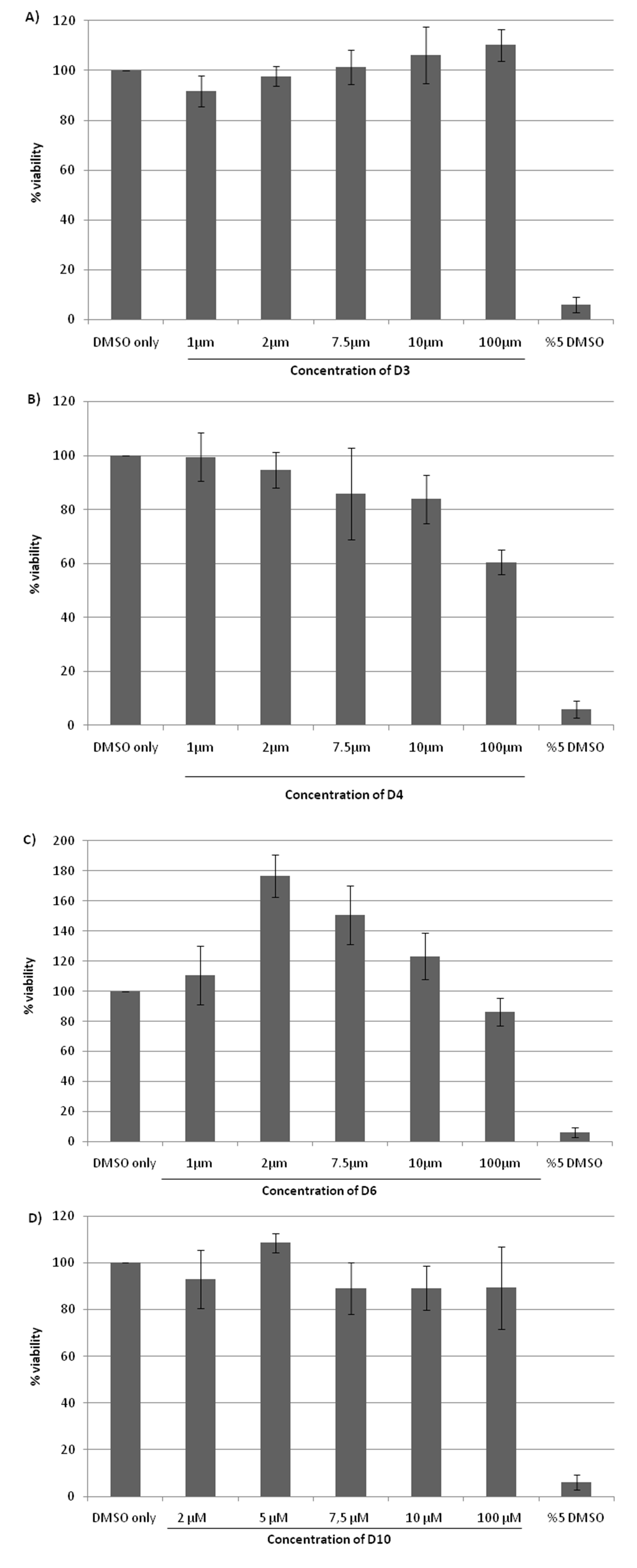

Supplement: Figure S5 — Measurement of the viability of HELA cells in the presence of ranging concentrations of D3 (A), D4 (B), D6 (C) and D10 (D)from 1 to 100 µM. Data are mean ±SEM for 6 independent experiments (p<0.00001). (JPG) [file pone.0031787.s005.jpg]

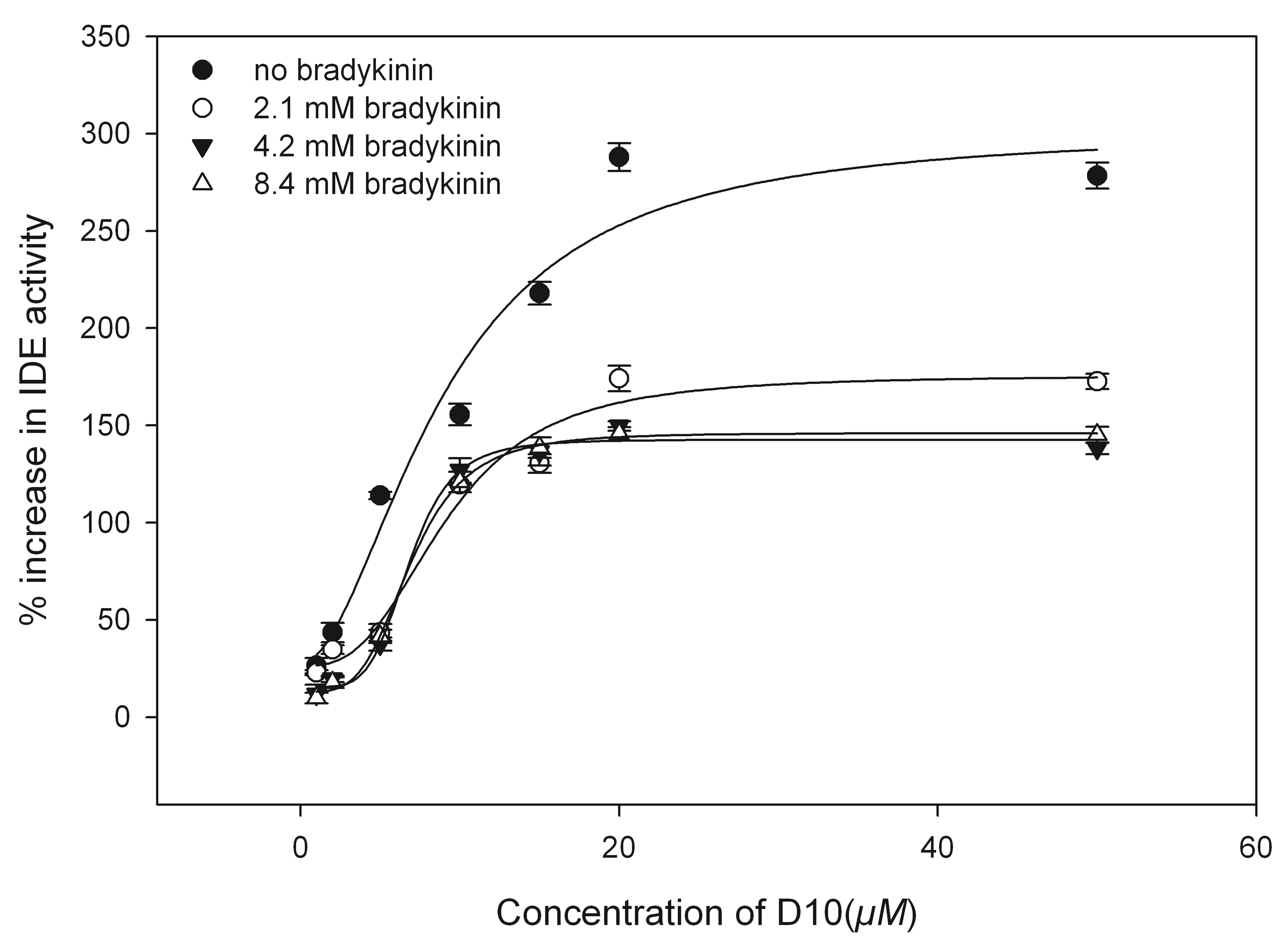

Supplement: Figure S6 — Competition of bradykinin with compound D10. Degradation of insulin in the presence of D10 is decreased by about 50% when there was bradykinin in the proteolytic solution. (JPG) [file pone.0031787.s006.jpg]

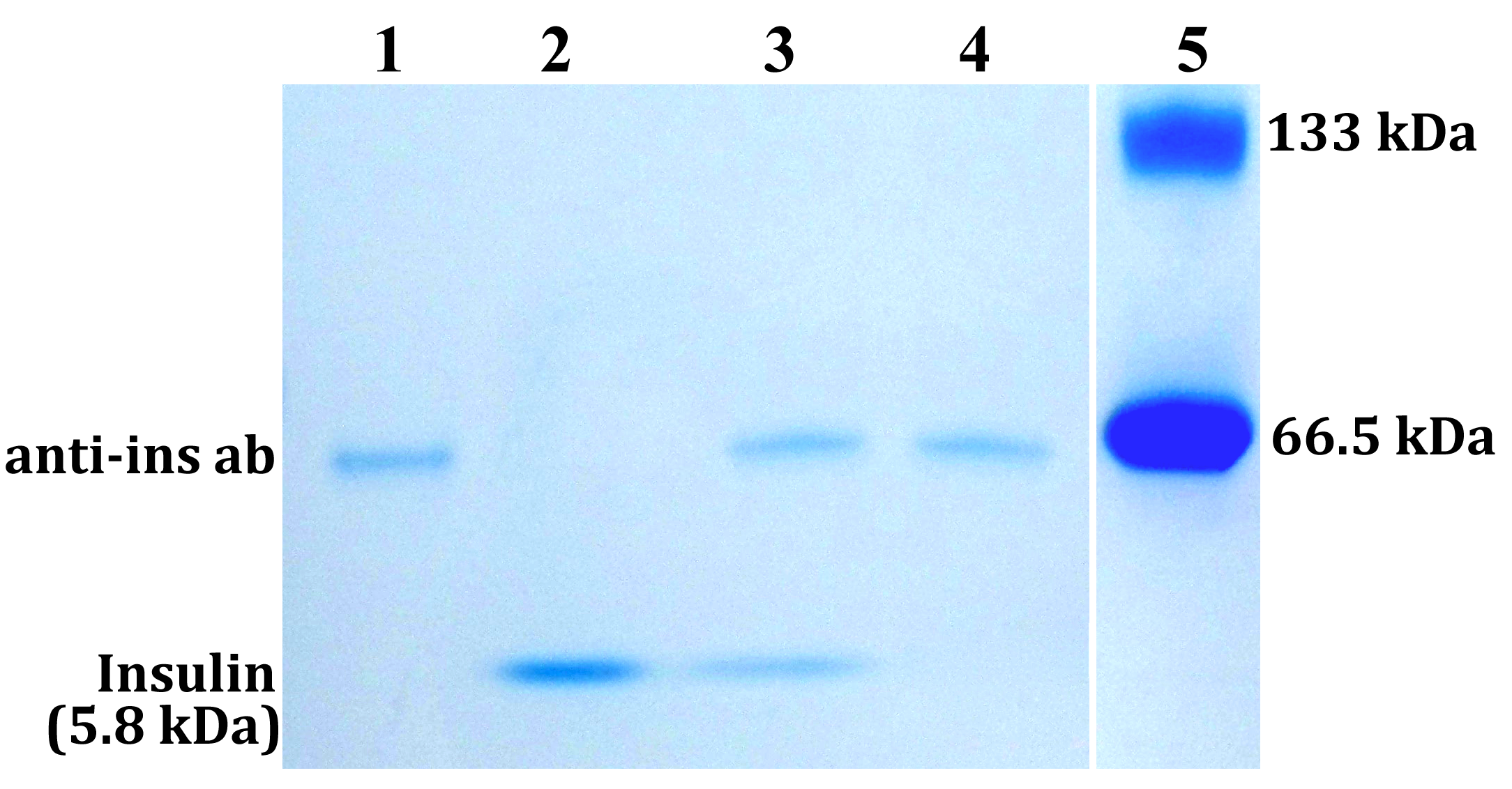

Supplement: Figure S7 — Binding of monoclonal antibody (mAb) to insulin. Lane 1: 400 nM mAb, lane 2: 400 ng insulin, lane 3: 400 nM mAb and 400 ng insulin, lane 4: 400 nM mAb and 200 ng insulin, lane 5: 1 mg/ml BSA. (TIF) [file pone.0031787.s007.tif]

**Figure S8.** Molecular structure of the compounds.

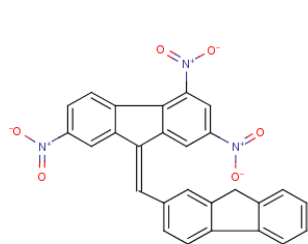

D1

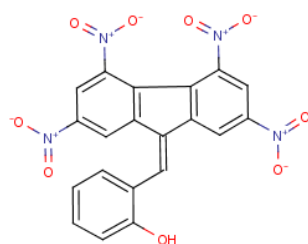

D2

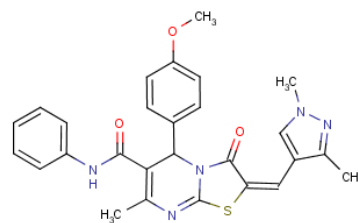

D3

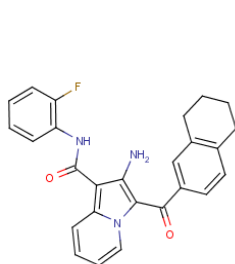

D4

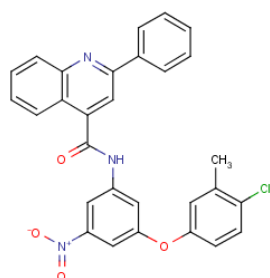

D5

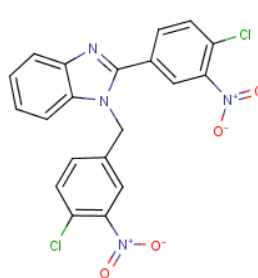

D6

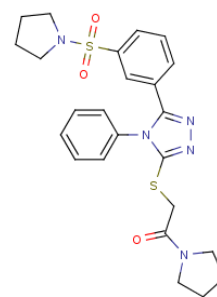

D7

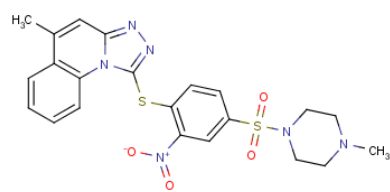

D8

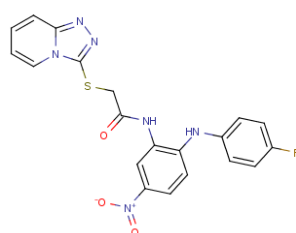

D9

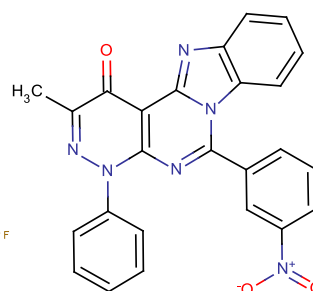

D10

Supplement: Figure S8 — Molecular structure of the compounds. (PDF) [file pone.0031787.s008.pdf]
